# Interventions to improve cognitive performance in chronic kidney disease: A scoping review

**DOI:** 10.1371/journal.pone.0329815

**Published:** 2025-08-07

**Authors:** Janine Farragher, Urooj K. Khan, Kevin Yau, Katherine E. Stewart, Tyrone G. Harrison, Lisa Engel, Samantha E. Seaton, Maoliosa Donald, Brenda R. Hemmelgarn

**Affiliations:** 1 Department of Occupational Science and Occupational Therapy, University of Toronto, Toronto, Canada; 2 Rehabilitation Sciences Institute, University of Toronto, Toronto, Canada; 3 Temerty Faculty of Medicine, University of Toronto, Toronto, Canada; 4 Department of Medicine, University of Calgary, Calgary, Canada; 5 Department of Community Health Sciences, University of Calgary, Calgary, Alberta, Canada; 6 O’Brien Institute for Public Health, Cumming School of Medicine, University of Calgary, Calgary, Alberta, Canada; 7 Libin Cardiovascular Institute, Cumming School of Medicine, University of Calgary, Calgary, Alberta, Canada; 8 Department of Occupational Therapy, University of Manitoba, Manitoba, Canada; 9 Institute for Work and Health, Toronto, Canada; 10 Faculty of Medicine and Dentistry, University of Alberta, Alberta, Canada; University of Luzon, PHILIPPINES

## Abstract

**Rationale & objective:**

Cognitive impairment is commonly associated with chronic kidney disease (CKD. A number of intervention approaches have the potential to improve cognitive performance in CKD. Our objective was to characterize interventions studied to improve cognitive performance for adults with CKD across all categories of severity, including kidney failure.

**Study design:**

Scoping review following JBI methodology.

**Setting and study populations:**

Adults (≥18 years) with CKD or kidney failure.

**Selection criteria for studies:**

We searched 5 electronic databases for studies published up to April 5, 2024. Eligible sources were primary research studies that investigated any intervention targeting cognition in adults (≥18 years) with CKD or kidney failure. Full-text article screening was performed in duplicate.

**Data extraction:**

Characteristics of interventions, populations studied, and outcomes investigated.

**Analytical approach:**

Descriptive statistics and narrative syntheses.

**Results:**

Seventy-one studies were included. Over half (n = 37, 52%) were conducted within the past five years, and most studies (n = 47, 66%) targeted people on maintenance hemodialysis therapy. Just over one-third of studies investigated pharmacological interventions, with much of the pharmacological or medical research focusing on anemia management or dialysis adequacy. Although recent research has expanded in focus, many other purported mechanisms of cognitive dysfunction in CKD remain understudied in interventional research. Exercise training (n = 14) was the most common nonpharmacological approach studied, but few studies have explored other promising nonpharmacological approaches such as cognitive rehabilitation interventions.

**Limitations:**

Abstract screening not performed in duplicate; non-English studies excluded.

**Conclusion:**

Research into cognitive interventions for people with kidney disease has primarily focused on the hemodialysis population and investigated erythropoietin stimulating agents, frequent or prolonged dialysis, and exercise, although there has been recent growth of research activity into other interventions. Future research should aim to address a broader range of purported pathophysiological mechanisms of cognitive impairment in CKD, investigate interventions for predialysis and peritoneal dialysis patients, and explore the impacts of established cognitive rehabilitation approaches.

## Introduction

Cognitive impairment is a common complication of chronic kidney disease (CKD) [1,2] that has been recognized as a serious public health issue [1]. Characterized by changes or declines in cognitive functions such as memory, executive functions, or judgment/decision making, an estimated 20–50% of people with chronic kidney disease (estimated glomerular filtration rate (eGFR) <60 mL/min/1.73 m² for >3 months or presence of albuminuria) experience cognitive impairment [3], which often worsens as CKD progresses [4] and affects approximately 50% of people with kidney failure (eGFR < 15 mL/min/1.73 m^2^) on hemodialysis and 40% of people on peritoneal dialysis [5]. However, cognitive impairment has historically been underrecognized and unaddressed in kidney disease, with one landmark multicenter study from 2004 finding that only 5% of patients on hemodialysis had ever undergone cognitive screening and/or referral to an appropriate specialist [1]. Although developments in certain parts of the world (e.g., mandated cognitive screening for Medicare Part B beneficiaries during annual wellness visits in the U.S.A.) have since aimed to improve detection of cognitive issues, gaps in screening protocols in both CKD and dialysis clinics remain. Potential barriers include time constraints in busy clinical settings, lack of staff training, and uncertainty about which tools are appropriate and clinically meaningful in this population, while patient fatigue, sensory impairments, and the fluctuating cognitive state associated with the dialysis cycle also complicate both the timing and interpretation of assessments [6,7].

Due to the under-recognition of cognitive challenges in this population,interventions that can address cognitive impairment in CKD are unclear. Unaddressed cognitive dysfunction can lead to functional dependence, reduced quality of life, caregiver burden, and long-term care admission [8–11], health outcomes that have been identified as top priorities of people living with CKD. Severe cognitive impairment or dementia among people with kidney failure on hemodialysis is also associated with an approximately 2-fold increased risk of mortality [12], further emphasizing its profound impact on patients. Investigating the range of interventions with the potential to reduce cognitive dysfunction and promote quality of life for people with CKD should thus be a priority for research.

**Fig 1:**
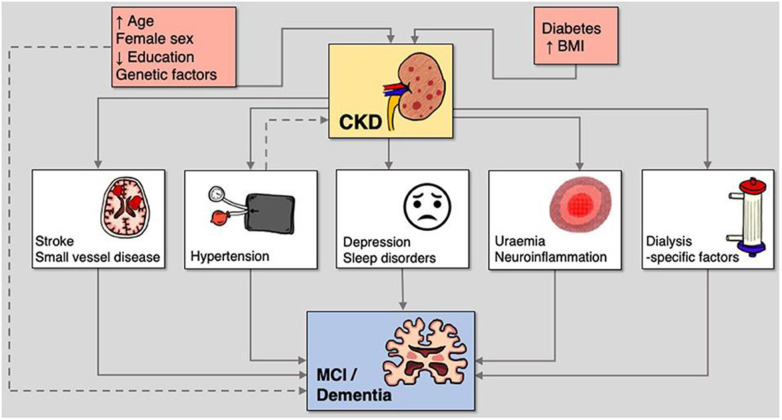
Mechanisms linking chronic kidney disease to cognitive impairment. Adapted from Kelly & Rothwell (2022), *Frontiers in Neurology*, under CC BY 4.0 license.

In CKD, the underlying mechanisms of cognitive impairment are believed to be multifactorial and complex [6,13] (Fig 1). People with CKD often have traditional cardiovascular risk factors such as diabetes and hypertension, which can contribute to the risk for cerebrovascular disease [6,14]. Uremic metabolites begin to accumulate in CKD before the onset of kidney failure and increase as kidney function declines, and while high-flux, high-efficiency dialysis membranes reduce severe uremic encephalopathy in those on dialysis, many middle molecules are inadequately cleared by conventional dialysis and could have deleterious effects on cognitive functioning [6,15] Anemia is a common complication of CKD that has been associated with cognitive impairment in some studies [6,13], while polypharmacy is common in people with CKD who frequently receive medications that may affect cognition, such as opioids, psychotropics, antivirals, and drugs with anticholinergic properties [16]. Depression and sleep disorders are also highly prevalent in people with CKD and may further interfere with cognitive functioning [6], while factors such as electrolyte disturbances, hyperparathyroidism, and vitamin D deficiencies might also contribute to cognitive deficits [13]. Importantly, hemodialysis treatment can induce hemodynamic instability and itself lead to ischemic cerebral injury in those with kidney failure [17,18]. Given its complexity, the optimal management of cognitive impairment in people with CKD should be holistic, seeking to prevent, mitigate or reverse its underlying mechanisms while also supporting people with cognitive impairments and their caregivers using evidence-based approaches to maximize their quality of life.

A variety of interventions to mitigate cognitive dysfunction in CKD have previously been proposed. These include addressing cardiovascular risk factors; using ACE, ARBs, and lipid-lowering drugs; improving blood pressure control; treating depression; improving sleep; and implementing medication deprescribing protocols [6,13,15,16]. For those on dialysis, improving dialytic clearance and using intradialytic cooling have also been proposed [6,13,15,16]. The International Federation on Ageing Think Tank on Dementia further recommends nonpharmacological approaches to support people with cognitive impairments, including addressing its impact on disability and functioning (e.g., through physical or cognitive rehabilitation programs); employing targeted rehabilitation interventions after acute illness or injury; supplying assistive technology to aid functioning; and providing caregiver support and education [19]. Each of these approaches represent potential opportunities to improve outcomes for people with CKD and cognitive impairment, and thus warrant exploration in the research literature.

Given that cognitive impairment has been historically underappreciated in kidney disease [20], it is unclear to what extent the range of interventions to support patients in optimizing their cognitive functioning and quality of life have been explored. The primary objective of this scoping review was therefore to identify and describe interventions studied to improve cognitive performance across the spectrum of chronic kidney disease, with a view to identify key knowledge gaps and opportunities for further research and clinical intervention.

## Materials and methods

We used scoping review methodology, as it enables a broad and comprehensive examination of literature to identify knowledge gaps, clarify concepts, and inform directions for future research [21]. Our review was guided by the JBI guidance on scoping reviews [22], follows a published protocol [23], and adheres to the reporting guidelines from the PRISMA Extension for Scoping Reviews (PRISMA-ScR) [24].

### Article eligibility criteria

#### Types of participants.

Eligible studies investigated adults (≥18 years) with CKD (defined as an estimated GFR < 60mL/min/1.73m^2^ for >3 months or presence of albuminuria [25]), including adults with kidney failure treated with all dialysis modalities. Studies focusing on children or people living with kidney transplants were excluded, as their needs for cognitive interventions may be distinct from patients with CKD or kidney failure on dialysis.

#### Concept.

Studies were included if they investigated an intervention stated to be targeting cognitive functioning, broadly defined as all forms of knowing and awareness (e.g., perceiving, conceiving, remembering, reasoning, judging, imagining, and problem solving [26]). Studies were also included if the intervention targeted one or more specific cognitive domains included in the “mental function” component of the World Health Organization International Classification of Functioning [27] inclusive of consciousness, orientation, intellectual function, attention, memory, perception, thought functions, higher-level cognition, mental functions of language, calculation, and/or experience of self and time. In addition, studies were included if they investigated the effect of an intervention on cognition or more than one of the aforementioned cognitive domains, irrespective of the inherent nature or focus of the intervention. Studies were excluded if they investigated interventions not specifically targeting cognitive functioning, such as cognitive-behavioural interventions designed to target mood or psychosocial well-being, but not cognition, or self-management interventions not aimed at specifically addressing cognitive impairment.

#### Context.

Studies from any year, countries, or practice settings were considered. Studies that were not available in English were excluded.

#### Types of sources.

Information sources included full-text, primary research articles. Primary studies using qualitative, quantitative, or mixed methods designs (including randomized controlled trials, quasi-experimental studies, pre-post studies, observational studies, pilot studies, single-case experiments, qualitative studies) were included, with no limits placed on publication date. We excluded case series, case studies, clinical practice guidelines, theoretical papers, theses and opinion-driven reports (editorials, non-systematic or literature/narrative reviews). Reference lists of relevant scoping and systematic reviews from the initial search were examined to identify additional articles.

#### Search strategy & article selection.

**Fig 2:**
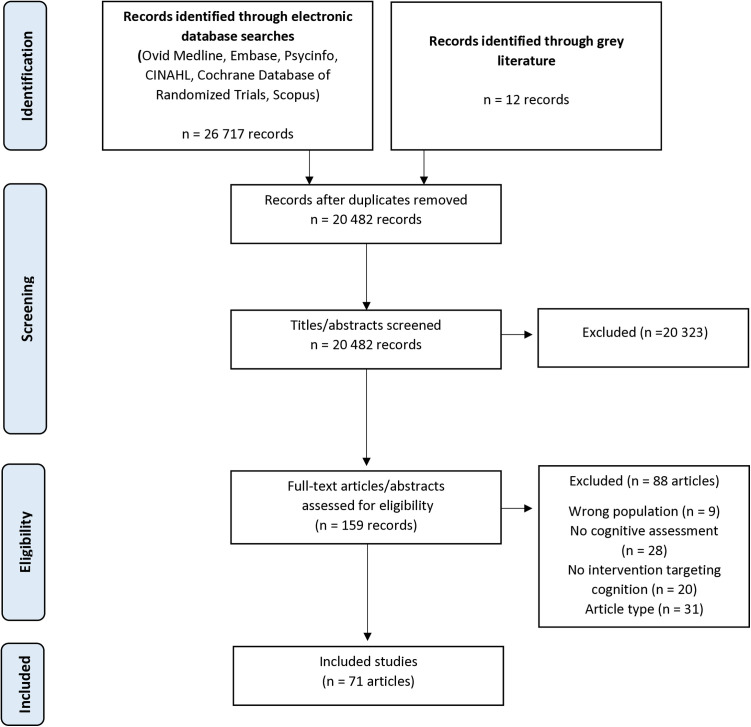
PRISMA Diagram of Study Selection Process.

The screening process is outlined in the PRISMA diagram in Fig 2. We worked with an information specialist to select search terms that represent the target population (CKD), concept (cognition), and intervention (Table 1). Our selection of cognition search terms was informed by prior reviews on cognitive interventions [28,29]. We searched the following six electronic databases up to April 2024 to identify relevant literature: Medline (OVID), EMBASE, PsycINFO, Cochrane Central Register of Controlled Trials, CINAHL Plus, and SCOPUS. A search was also conducted using the Canadian Agency for Drugs and Technologies in Health (CADTH) guidelines to search online search engines (Google Canada/US/UK), relevant Health Technology Assessment agencies, and Clinical Trials databases. In addition, we performed backward citation searching, examining reference lists of included studies and relevant systematic/scoping reviews to identify literature.

**Table 1:** Article characteristics.

	Author	Year	Study Design	Sample size	Intervention	Intervention description	Control	Cognitive Outcomes	Effect Trend
ALL CKD (PREDIALYSIS + DIALYSIS)									
PHARMACOLOGICAL									
[31]	Singh	2006	Pre-post	30	rHuEPO therapy	rhuEPO 100 IU/kg subcutaneous twice a week for 6 weeks.	None	Cognitive event-related potentials	+
[32]	Liu	2016	Observational	11,943	Influenza vaccination	At least 1 influenza vaccination	None	Incidence of dementia	+
[33]	Brady	2009	Secondary analysis of RCT	659	High-dose Vitamins	Daily doses of folic acid (40 mg), pyridoxine (vitamin B6; 100 mg) and cyanocobalamin (vitamin B12; 2 mg)	Placebo	Global	–
								Memory	–
NONPHARMACOLOGICAL									
[34]	Gronewald	2016	Observational	173	Dedicated university nephrology care environment	NR	None	Global	+
								Memory	+
								Processing speed	+
								Verbal fluency	+
PREDIALYSIS ONLY									
PHARMACOLOGICAL									
[35]	Revicki	1995	RCT	83	rHuEPO therapy	50 U/kg r- HuEPO subcutaneously, three times a week. When patients reached a HCT of 36%, the drug dosage was titrated to maintain a HCT of 35%.	Usual care	Global	–
[36]	Alexander	2007	Pre-post	62	rHuEPO therapy	Starting dose of 0.45 µg/kg once-weekly for 24 weeks. Dose adjusted to maintain a Hb rise between 1.0 and 3.0 g/dL over 4 weeks until 12.0-13.0g/dL was achieved	None	Self-reported	–
[37]	Chen	2021	Retrospective cohort study	21,379	Angiotensin receptor blockers	Any ARB use during the exposure period	No ARB use during exposure period	Incidence of dementia	+
[38]	Mone	2021	Retrospective case-control	41	Rivastigmine	Not specified	Mild cognitive impairment patients without CKD on rivastigmine	Global	+
[39]	Cha	2022	RCT	150	AST-120	AST-120 orally in three divided doses for a total of 6 g a day for 48 weeks	Standard care	Self-reported cognition	+
[40]	Johansen	2022	RCT	614	Daprodustat	Daprodustat dosed daily and titrated to maintain Hb 11–12 g/dl for 28 weeks	Placebo	Self-reported cognition	+
[41]	Kendrick	2023	RCT	109	NaHCO3	NaHCO3 at a dose of 0.5 mEq/LBW-kg/day for 12 months	Placebo	Global	–
[42]	Jong	2023	Retrospective cohort study	55,162	Statin use	Statin prescription	No statin prescription	Incidence of dementia	–
NONPHARMACOLOGICAL									
[43]	Otobe	2021	RCT	60	Exercise training	60 mins of group exercise once per week and home exercises twice per week for 24 weeks	Usual care	Global	–
								Memory	+
								Attention	–
								Executive function	–
								Verbal fluency	–
[44]	Bronas	2021	RCT	39	Home-based walking program	Home based walking for 20–60 mins 3–5 times per week for 6 months	Physical activity advice	Global	–
[45]	Bronas	2022	Protocol	34	Walking exercise program	20-60 mins of moderate intensity walking, 3 times per week for 24 weeks	Usual care	Global	TBD
								Memory	TBD
								Executive function	TBD
								Verbal fluency	TBD
[46]	Campbell	2008	RCT	60	Individualised nutritional counselling	An initial individual consultation with a dietitian, then telephone consultation fortnightly for the first month, then monthly over 12 weeks	Standard care (written material on generic nutrition information for CKD and co-morbidity management)	Self-reported cognition	+
ALL DIALYSIS (HD + PD)									
PHARMACOLOGICAL/MEDICAL									
[47]	Mathur	2022	Retrospective cohort study	189,433	Treatment for secondary hyperparathyroidism	Vitamin D analogs, phosphate binders, cinacalcet, and/or parathyroidectomy	No treatment for secondary hyperparathyroidism	Incidence of dementia	+
NONPHARMACOLOGICAL									
[48]	Loos-Ayav	2007	Observational	277	Self-care dialysis	Training program, then routine self-care dialysis	Conventional in-center HD or PD with nurse assistance	Self-reported	+
[49]	Manfredini	2017	RCT	296	Personalized, home-based, low-intensity walking program	Walking to a metronome for 10 mins twice per day, 3 days per week for 24 weeks	Usual care and generic advice to maintain an active lifestyle	Self-reported	+
[50]	Baggetta	2018	Secondary analysis of Manfredini (2017)	115	Personalized, home-based, low-intensity walking program	Walking to a metronome for 10 mins twice per day, 3 days per week for 24 weeks	Usual care (received generic advice to maintain an active lifestyle)	Self-reported	+
HEMODIALYSIS									
PHARMACOLOGICAL/MEDICAL									
*rHuEPO therapy *									
[51]	Grimm	1990	Pre-post	16	rHuEPO therapy	70 + /- 15 U/kg thrice weekly at start, ramping up to 94 + /- 27 U/kg	6 HD patients without rHuEPO	Global	–
								Cognitive event-related potentials	+
[52]	Brown	1991	Pre-post	14	rHuEPO therapy	Not specified	None	Memory	+
								Executive function	+
								Cognitive event-related potentials	–
[53]	Marsh	1991	Pre-post	24	rHuEPO therapy	Not specified	None	Memory	Mixed
								Attention	+
								Executive function	Mixed
								Processing speed	+
								Verbal fluency	–
								Cognitive event-related potentials	–
[54]	Pickett	1999	Pre-post	20	rHuEPO therapy	rHuEPO increased until target Hct was achieved (mean, 42.8%)	None	Cognitive event-related potentials	Mixed
[55]	Vinothkumar	2019	Pilot study	60	rHuEPO therapy	rHuEPO, 100 IU/kg for 6 months, two times per week	None	Global	+
								Memory	+
								Executive function	+
[56]	Hung	2019	Retrospective cohort study	43,906	rHuEPO therapy; intravenous iron	NR	Usual care	Incidence of dementia	+
*L-Carnitine *									
[57]	Ueno	2021	Quasi-experiment	24	L-carnitine	Long-term l-carnitine treatment, 600 mg orally once per day for 2 years, then 1000 mg intravenously per hemodialysis day	No or short-term l-carnitine treatment	Global	–
								Memory	–
								Executive function	+
[58]	Atilgan	2021	Quasi-experiment	73	L-carnitine	1000 mg of IV L-Carnitine after every HD session for 6 months	Usual care	Global	+
*Other *									
[59]	Samaei	2018	RCT	39	Valerian	1 valerian capsule (Sedamin 530 mg) 60 minutes before bedtime for one month. Valerian (*Valeriana officinalis*) is a herb used in traditional medicine for the treatment of insomnia.	Placebo capsules (Starch 50 mg) 60 min before bedtime for one month	Global	+
								Memory	–
								Attention	–
								Verbal fluency	–
[60]	Yiannopoulou	2019	Case series	5	Donepezil	Donepezil 5 mg per day, administered at least 5 hours before hemodialysis sessions	None	Global	+
								Memory	+
								Executive function	+
[61]	Lu	2021	RCT	50	Thiamine and folic acid supplementation	Thiamine 90 mg/day and folic acid 30 mg/day	Usual care	Global	+
[62]	Marzieh	2023	RCT	108	Melatonin before bed	Melatonin 3 mg tablet half an hour before bed at night every night for 6 weeks	Placebo tablet	Global	+
[63]	Lin	2023	Retrospective cohort study	1424	Vitamin D	Vitamin D prescription	No Vitamin D prescription	Incidence of dementia	+
DIALYSIS-SPECIFIC									
*Hemodiafiltration *									
[64]	Jaber	2004	Pilot study	14	Daily hemodiafiltration	Daily hemodiafiltration (Monday through Saturday) for 4 weeks	None	Self-reported	+
[65]	Wang	2022	Quasi-experiment	44	Hemodiafiltration	Hemodialysis three times per week, including hemodiafiltration session twice per month	Conventional hemodialysis	Global	–
								Memory	–
								Attention	–
								Executive function	–
								Processing Speed	–
								Verbal fluency	+
[66]	Jimenez	2024	Pilot study	24	Hemodiafiltration	One hemodiafiltration session lasting 2 hours	Conventional hemodialysis	Global	Mixed
*Frequent/prolonged dialysis *									
[67]	Jassal	2006	Pilot study	14	Frequent nocturnal hemodialysis	6 and 10h each night; 5–7 times a week	None	Memory	+
								Attention	+
								Processing speed	+
[68]	Ok	2010	Quasi-experiment	494	Prolonged nocturnal dialysis	3 sessions per week for 7–8 hours per night	Conventional thrice-weekly hemodialysis	Global	–
								Executive function	–
[69]	Rocco	2011	RCT	118	Frequent home nocturnal hemodialysis	Dialysis prescriptions subject to a std Kt/Vurea of X4.0 and a treatment time of 6h or more, 6 days per week	Usual three times per week hemodialysis prescription, subject to a prescribed eKt/V urea 41.1, a std Kt/V urea of42.0, and a treatment time X2.5 h/ session	Global	–
[70]	Kurella Tamura	2013	RCT	245 (daily trial), 87 (nocturnal trial)	Frequent hemodialysis	6 times per week for 1.5–2.75 hours (DT);6 times per week for 6–8 hours (NT)	Conventional thrice-weekly hemodialysis	Global	–
								Memory	Mixed
								Attention	–
								Executive function	–
								Processing speed	–
[71]	Dixon	2016	Observational	77	Frequent nocturnal hemodialysis	≥6 h per night, 6 nights per week to achieve a weekly target stdKt/V ≥ 4.0/week	None	Global	–
								Memory	Mixed
								Attention	–
								Processing speed	–
*Other *									
[72]	Dokugan	2009	Quasi-experiment	66	Strict volume control	Hemodialysis for 4h three times weekly, using bicarbonate dialysis fluid and synthetic polysulfone membranes with a surface area of 1.6 m2. The blood flow was 250–300 mL/min, and the dialysate flow was 500 mL/min. Ultrafiltration was controlled volumetrically.	Healthy controls; dialysis patients on antihypertensive drugs	Cognitive event-related potentials	+
[73]	Hannestad	2020	Protocol	26	AKST1210 (a device that removes beta-2-microglobulin from plasma)	NR	None	Global	TBD
[74]	Kaja Kamal	2020	Protocol	180	Incremental hemodialysis	Following randomisation dialysis will be initiated twice weekly, with a session duration of 3.5–4 hours. Dialysis frequency adjusted to meet adequacy targets.	Conventional thrice-weekly hemodialysis	Global	TBD
[75]	Dasgupta	2020	Protocol	90	Cooled dialysate	Dialysate temperature of 35 degrees C	Standard dialysate temperature of 36.5 degrees C	Global	TBD
NONPHARMACOLOGICAL									
*Exercise *									
[76]	Poorsaadet	2018	RCT	38	Intradialytic aerobic exercise	Intradialytic aerobic exercise 3 times per week for 75 minutes during the first 2 hrs of dialysis for six months	Control (not otherwise described)	Global	+
[77]	Stringuetta- Belik	2018	Pilot study	35	Intradialytic aerobic exercise	Intradialytic aerobic exercise 3 times per week for 4 months	Usual care	Global	+
[78]	Grigoriou	2021	Pre-post	20	Long-term intradialytic hybrid exercise training	60-80 mins of intradialytic aerobic and resistance exercise, 3 times per week for 9 months	None	Global	+
[79]	Myers	2021	RCT		Home-based exercise program	1-3 supervised exercise sessions, followed by 12 weeks of home-based aerobic and resistance exercise for 45 mins per day	Usual care	Not specified	–
[80]	Nakamura-Taira	2021	Quasi-experiment	42	Resistance exercise training	25-30 mins of resistance exercise three times per week for 6 months	Stretching	Global	–
[81]	McAdams-DeMarco	2018	RCT	23	Exercise training or cognitive training	20 minute sessions at every dialysis session for 3 months	Standard care	Global	–
								Executive function	+
[82]	McAdams-DeMarco	2020	Protocol	200	Exercise training and cognitive training	30 mins of intradialytic bike exercise; 30 mins of lumosity brain games, 3 times per week for 3 months	Usual care	Global	TBD
								Memory	TBD
								Executive function	TBD
[83]	Bogataj	2023	RCT	44	Combined physical and cognitive intradialytic training	Three sessions per week for 12 weeks of aerobic exercise session via ergometer lasting 40 minutes, followed by tablet-based cognitive training on Cognifit lasting 30–40 mins	Standard care	Attention	Mixed
[84]	Kren	2023	RCT	44	Combined physical and cognitive intradialytic training	Three sessions per week for 12 weeks of aerobic exercise session via ergometer lasting 40 minutes, followed by tablet-based cognitive training on Cognifit lasting 30–40 mins	Standard care	Global	Mixed
*Cognitive/Educational *									
[85]	Ahmadzadeh	2017	Pre-post	53	Chronic disease self-management program	5 weeks; other details not specified	None	Self-reported	–
[86]	Erdley-Kass	2018	RCT	33	Problem-solving training	Six 1-hour weekly treatment sessions	Usual care	Problem-solving orientation	Mixed
[87]	Noguchi	2020	Pre-post	12	N-back cognitive training	N-back training (working memory tasks on an iPad) 20 mins per hemodialysis session, 3 times per week for 3 months	None	Global	+
								Memory	+
								Attention	Mixed
								Executive function	+
[88]	Ren	2022	Quasi-experiment	80	Self-management micro-video health education program	3-month micro-video health education program, with each video lasting 10 mins. One-on-one health education was conducted during each 2-week follow-up.	Standard care	Self-reported	+
[89]	Keivan	2023	RCT	60	Self-management program using 5A nursing model	Self-management program based on the 5 A model, which was implemented in five stages through face-to-face meetings, phone calls,and SMSs over three months	Standard care	Self-reported	–
[90]	Xia	2024	Quasi-experiment	92	Teach-back health education strategy	Teach-back health education about hemodialysis self-management administered biweekly for 60–90 mins over 6 months	Conventional health education	Self-reported	+
*Generalized Rehabilitation *									
[91]	Endo	2017	Observational	182	Daily rehabilitation	Daily rehabilitation for 2–178 days (mean 74 days) for 20 mins (dialysis days) or 60 mins (non-dialysis days)	None	Functional cognition	–
[92]	Farragher	2020	Retrospective cohort study	449	Geriatric dialysis rehabilitation program	Intensive inpatient rehabilitation program for older HD patients. Key feature was scheduled short daily HD 6 days per week.	None	Functional cognition	–
Other									
[93]	Stumm	2019	Pre-post	63	Educational intervention about hyperphosphatemia	40 mins of individual guidance during hemodialysis sessions for 60 days. Included verbal guidance about CKD, signs and symptoms of hyperphosphatemia, use of phosphorus chelators, nutrition, and alternatives to improve HRQoL	None	Self-reported cognition	+
[94]	Suzuki	2019	Protocol	20	Electrical muscle stimulation	EMS performed on each leg using belt electrode skeletal EMS for 30–40 mins per day, 3 days per week for 5 weeks during hemodialysis	Usual care	Global	N/A
[95]	Lai	2020	Quasi-experiment	41	Low protein diet	Low-protein diet for 6 months, developed by an expert dietician and personalized for each patient	Usual care	Global	+
[96]	Dehghan	2021	RCT	110	Lavender, rosemary and orange essential oils	Gauze containing essential oils placed 10–15 cm from patients nose 1 hr after HD sessions for 30 mins, 3 times per week for 1 month	Usual care	Memory	–
[97]	Ameri	2021	RCT	75	Reminiscence therapy	Two 30 min group reminiscence sessions per week for 6 weeks, focused on reminiscing about past memories about their lives	Group discussion (sham)	Global	+
[98]	Vaishnav	2022	RCT	80	Guided meditation	Guided meditation 30 mins 3 times per week during dialysis for 6 weeks	Standard care	Self-reported cognition	–
[99]	Attiya	2023	RCT	50	Remote ischemic preconditioning	Sphygmomanometer cuff placed around non-vascular access arm before each HD session for 12 weeks. Three cycles of ischemia for five minutes performed followed by reperfusion for five minutes. Ischemia induced by inflating sphygmomanometer cuff to 200 mmHg.	Standard care	Global	–
PERITONEAL DIALYSIS									
PHARMACOLOGICAL									
[100]	Temple	1995	Quasi-experiment	17	rHuEPO therapy	rHuEpo (epoetin alfa, Eprex, Cilag Ltd) in a starting dose of 50 units/kg s.c. twice weekly	Usual care	Memory Attention Processing speed	–
									+
									–
NONPHARMACOLOGICAL									
[101]	Lee	2022	RCT	23	Virtual reality program for PD training	Eight sessions of VR training on PD exchanges, 1–2 times per week	Print and educational materials on PD training	Memory Executive function	–
									–

Following the initial search, results were imported into Rayyan [30], an online tool for eligibility screening. Screening was conducted independently by three reviewers after excellent inter-rater agreement (κ = 0.92) was established for 10% of the titles/abstracts. Full-text screening was conducted independently in duplicate for all articles. Disagreements about eligibility were resolved by discussion with a third reviewer to obtain consensus.

#### Charting, collating and summarizing data.

A data extraction spreadsheet was developed *a priori* and piloted with five articles before data extraction was undertaken. Once piloted, two reviewers performed independent data extraction for each included study, and disagreements were resolved by discussion to reach consensus. Data extracted included article characteristics (e.g., authors, date, journal, country, and study design), population characteristics (e.g., age, sex, CKD category and definition, and screening criteria), cognitive intervention characteristics (e.g., aims, theoretical background, design, dose/duration, materials, location, provider(s)), and study outcomes (e.g., outcomes assessed, outcome measures used, results reported). A narrative summary of the characteristics of the literature was completed, with counts and percentages used to describe patterns in the literature. Reported findings of intervention outcomes were categorized as positive, unchanged, or negative for each study to describe general trends, with no critical examination of evidence quality.

## Results

The initial search strategy generated 26,717 results. After duplicates were removed and articles were screened and selected for eligibility, 71 studies were included in the review (Fig 2).

### Study characteristics

**Fig 3:**
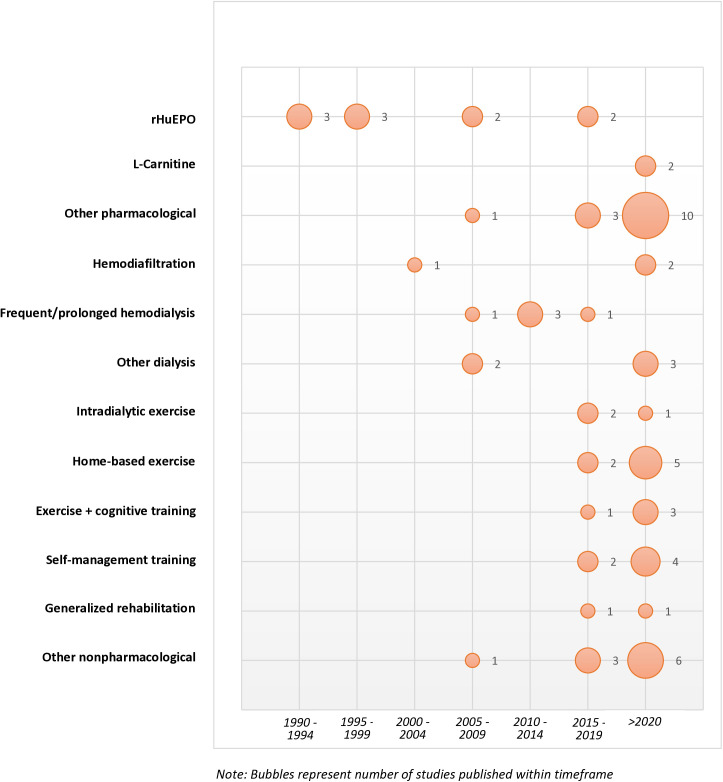
Historical Map of Interventions Studied for Cognitive Impairment in Kidney Disease.

Most studies were conducted recently, with more than half (n = 37, 52%) published since 2020 and 73% (n = 52) published within the last 10 years (Fig 3). Studies were most frequently conducted in Asia (n = 30, 42%), North America (n = 23, 32%) or Europe (n = 14, 20%), with two studies from South America, one study from Africa and one study from Australia. Most studies (n = 47, 69%) specifically targeted people with kidney failure on hemodialysis therapy. Thirty-seven percent of studies (n = 26) were randomized controlled trials, with a range of other study designs that include quasi-experiments (n = 10, 14%), uncontrolled pre-post studies (n = 10, 14%), retrospective cohort studies (n = 6, 8%), study protocols (n = 5, 7%), pilot studies (n = 5, 7%), observational studies (n = 4, 6%), a case-series study, a case-control study, and a secondary analysis of a randomized controlled trial. Cognitive functions were the primary outcome in 43 (61%) studies and a secondary outcome in 27 (39%) studies.

### Interventions

#### Dialysis.

**Fig 4:**
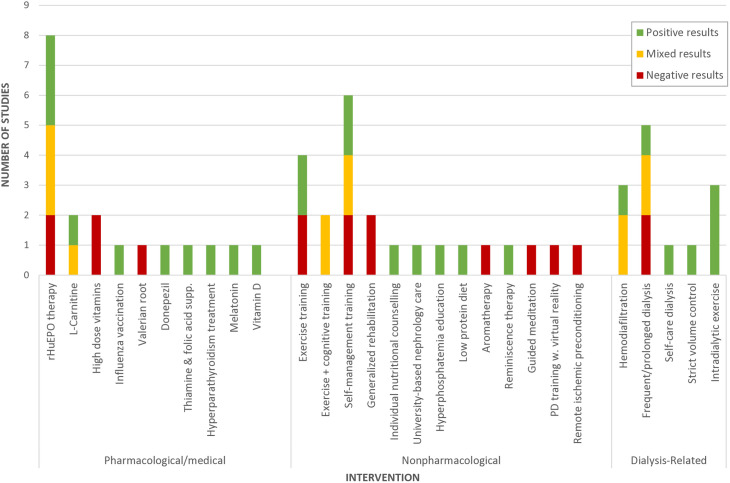
Cognitive Effect Trends Reported in Dialysis Intervention Studies.

The majority of studies (n = 49) exclusively targeted people on hemodialysis therapy, with only six studies including people on PD and only two studies specifically targeting the PD population. For the hemodialysis population, there were 13 studies of pharmacological interventions, 12 studies of dialysis-related interventions, and 24 studies of nonpharmacological interventions. Erythropoietin stimulating agents (ESAs) were the most frequently studied pharmacological approach (n = 6). The other seven studies investigated six different pharmacologic interventions, including two studies of L-carnitine, and one study each of donepezil, melatonin, vitamin D, thiamine and folic acid, and valerian. Dialysis-related interventions included more frequent hemodialysis (n = 5), hemodiafiltration (n = 3), cooled dialysate (n = 1), incremental hemodialysis (n = 1), AKST1210 beta-2-microglobulin removal (n = 1), and ultrafiltration with a low-salt diet (n = 1). Nonpharmacological interventions consisted of several types of exercise programs such as home or community-based exercise programs (n = 2), intradialytic exercise programs (n = 3), and exercise programs combined with computerized cognitive training activities (n = 4). Six studies explored self-management education programs, and two studies investigated generalized, multimodal inpatient rehabilitation programs. The remaining nonpharmacological approaches were diverse, including approaches such as remote ischemic pre-conditioning, virtual reality for PD training, guided meditation, and aromatherapy. The two studies investigating interventions for people on peritoneal dialysis included one study of rHuEPO therapy and one study of a virtual reality program to support PD training. The four studies including people on either type of dialysis examined home-based exercise programs (n = 2), treatment for secondary hyperparathyroidisim, and a self-care dialysis model. Cognitive outcome trends reported for each intervention are depicted in Fig 4.

#### Predialysis.

**Fig 5:**
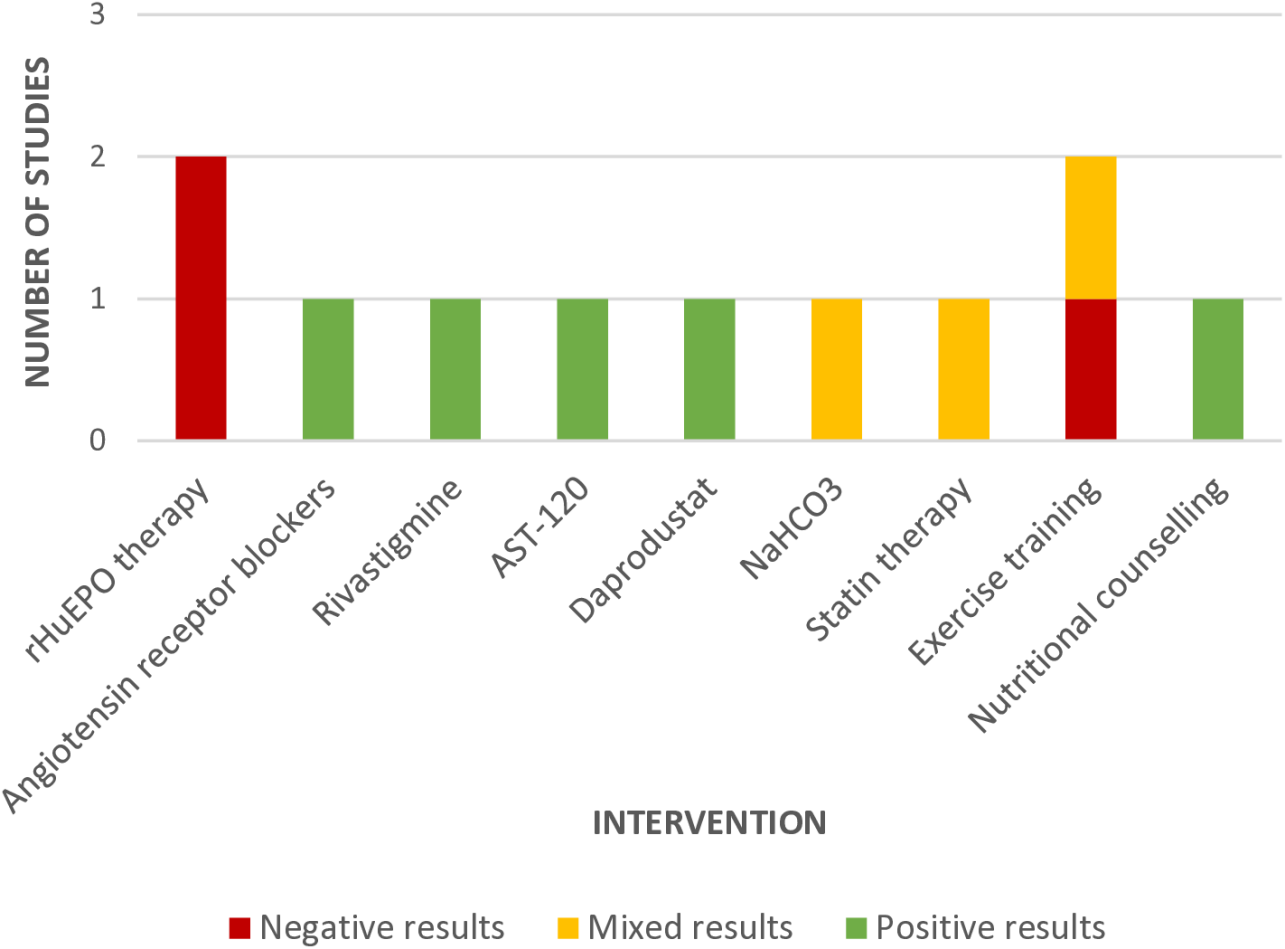
Cognitive Effect Trends Reported in Predialysis Intervention Studies.

Twelve studies investigated interventions to mitigate cognitive impairment for people with predialysis CKD, which included 8 pharmcological and 4 nonpharmacological interventions. Pharmacological interventions studied for this population included rHuEPO therapy, daprodustat, angiotensin receptor blockers, rivastigmine, AST120, NaHC03, and statin therapy. Nonpharmacological interventions included three light-intensity exercise programs, and one nutritional counselling intervention. See Fig 5 for an overview of cognitive outcome trends reported for each intervention.

### All CKD (Predialysis and Dialysis)

Four studies investigated interventions to mitigate cognitive impairment for predialysis and/or dialysis patients. The interventions included rHuEPO therapy, the influenza vaccination, high-dose vitamins, and a dedicated nephrology care environment.

### Outcome measurement

**Fig 6:**
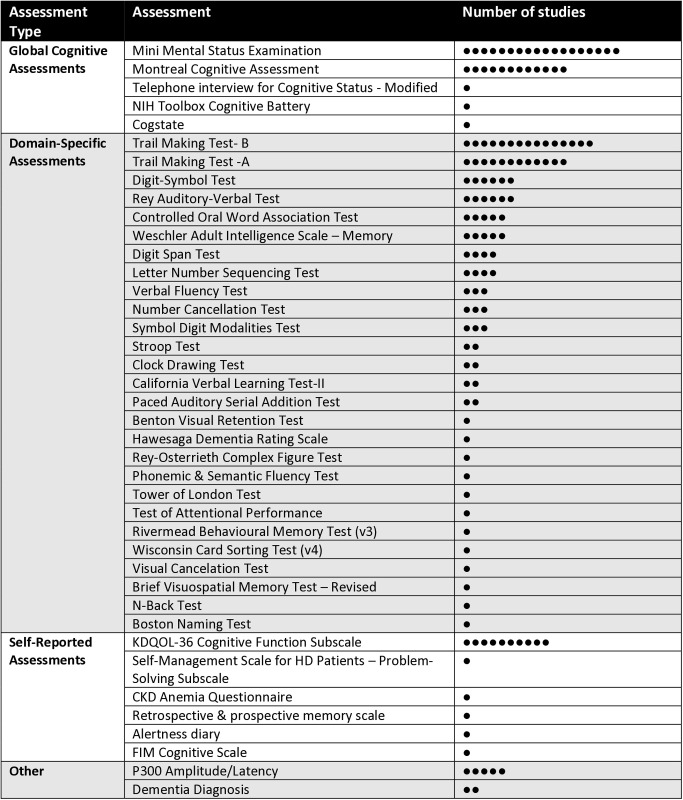
Cognitive Outcome Measures Used in Chronic Kidney Disease Research.

Most intervention studies (n = 43, 61%) measuring cognitive outcomes in CKD used one or more objective cognitive performance tests. Of these, seventeen studies used a global cognitive screening tool, with the MMSE (n = 19) and the MoCA (n = 12) the most commonly-used cognitive outcome measures. Twenty-five studies used one or more domain-specific neurocognitive tests, with memory (n = 19), executive function (n = 14), and attention (n = 10) the most frequently measured cognitive outcome domains. Fifteen studies assessed cognition subjectively, with the cognitive subscale from the KDQOL-36 the most commonly-used subjective cognitive tool (n = 10). Other subjective cognitive outcome measures included the problem-solving subscale from the self-management scale for hemodialysis patients (n = 2); the retrospective and prospective memory scale (n = 1); the cognitive subscale from the CKD anemia questionnaire (n = 1); and diary self-ratings of alertness, awareness and clarity of thought. No studies combined subjective cognitive measures with objective performance-based cognitive tests. Other cognitive outcome measurement approaches that were used include the incidence of dementia (n = 6), or were not specified (n = 3) (Fig 6).

## Discussion

In this review, we sought to characterize interventions that have been studied to address cognitive impairment in people with CKD. The majority of studies were preliminary and targeted people on hemodialysis, using a widely heterogeneous set of interventions among which ESAs, frequent or prolonged hemodialysis, and exercise training were the most common approaches. We found a relative dearth of research investigating cognitive interventions for people on peritoneal dialysis and people with predialysis CKD, and a widespread lack of research into supportive nonpharmacological interventions, such as cognitive rehabilitation, that can improve quality of life and participation in people with cognitive impairments [19,28,29,102]. We also found few studies incorporated patient perspectives in the design of interventions or captured subjective cognitive functioning as an outcome. Our findings collectively highlight a number of underexplored opportunities for supporting people in managing the cognitive complications of chronic kidney disease.

### Dialysis

A diverse set of pharmacological interventions have been studied for people on dialysis, which reflects the complex and multifactorial etiology of cognitive decline in this population [6]. That ESAs are the most widely-studied pharmacological approach for cognitive management reflects an early research boom into their impacts in the 1980’s, rather than strong potential to affect cognitive outcomes, which has not been conclusively demonstrated [6]. Outside of the ESA literature, there has recently been an increased exploration of alternative pharmacological strategies for cognitive management, but the evidence base remains largely preliminary. For example, we found few studies have targeted vascular contributions to cognitive impairment, despite strong evidence for cerebral small vessel disease in this population [103]. There have been no clinical trials or preliminary studies of approaches such as antihypertensives, oral anticoagulation therapies or antiplatelet therapies. While single studies addressed other possible mechanisms of cognitive impairment (e.g., electrolyte disturbances, hyperparathyroidism, vitamin D deficiency [41,47,61,63], this research is early-phase and larger trials with replication are needed. Meanwhile, pharmacologic interventions to target depression and sleep disorders have been neglected, and the cognitive impacts of reducing polypharmacy are unexplored. For the hemodialysis population, there has been some investigation into the cognitive impacts of interventions that reduce vascular insults caused by uremic metabolites and hemodynamic instability during hemodialysis. For example, increasing the frequency or duration of hemodialysis has received considerable attention, but studies in this area have generally not reported positive impacts on cognitive performance [67,69–71]. The impact of removing more middle-molecular weight solutes during dialysis through hemodiafiltration has also been explored, but evidence is currently based on small studies [64–66], and definitive trials are required. Cooled dialysate has also been proposed to address the circulatory stress associated with dialysis [6] with a previous study having demonstrated positive effects of cooled dialysate on brain white matter microstructure [104], but data showing an impact on cognitive performance has yet to be produced. Collectively, these findings highlight a number of knowledge gaps on pharmacological or dialysis-related cognitive management in the dialysis population.

The nonpharmacological literature in dialysis has been dominated by studies examining the impacts of exercise on cognitive function, which aligns with evidence from other populations that aerobic exercise can have a small beneficial effect on cognitive functioning [105,106]. Exercise also has other demonstrated benefits for people on dialysis [107,108], and there is little doubt that it should be viewed as a key aspect of care for this population. Family support, goal setting and accessibility of local facilities are important determinants of physical activity levels [109], while staff encouragement and support and use of a routine have also been identified as facilitators to intradialytic exercise [110]. Meanwhile, a focus on enjoyable activities, predictability, simplified instructions, and collaborative problem-solving around obstacles can help to engage people with cognitive impairments in physical activity [111]. Beyond exercise, there has been a widespread lack of research into other evidence-based nonpharmacological approaches for cognitive management in the hemodialysis population, such as cognitive rehabilitation, caregiver support, or environmental modifications. In particular, systematic reviews from multiple sclerosis, Alzheimer’s disease, and traumatic brain injury have outlined large evidence bases for cognitive rehabilitation interventions [19,28,29,102] but there has been almost no research into such approaches for people on dialysis. Such approaches often focus on promoting outcomes such as life participation and quality of life, which are prioritized by people living with kidney disease [112,113], and should thus receive greater focus in the research literature. We also note that no nonpharmacological intervention explicitly incorporated patient or caregiver input in their design or selection. This is concerning because individuals with cognitive impairment may face unique challenges with adherence, comprehension, and engagement [114,115] that can ultimately affect the feasibility and success of interventions. Caregivers often play a central role in managing these challenges, particularly in advanced CKD, and their perspectives can offer valuable insight into how cognitive changes impact daily life and care routines. Incorporating patient and caregiver voices is therefore essential to ensure that interventions are not only clinically effective, but also tailored to the practical realities, preferences, and priorities of those living with or supporting someone with cognitive impairment.

Despite a high prevalence of cognitive impairment in people on peritoneal dialysis, we found a wide discrepancy in the attention paid to cognitive management between hemodialysis and peritoneal dialysis, with only six studies in total including PD patients and just two studies examining cognitive interventions specifically for the PD population. PD patients face many of the same cognitive risks as those on HD, including toxin accumulation, vascular pathology, and inflammation [7,17], but also have distinct patterns of uremic toxin clearance, inflammatory burden, and oxidative stress, which may influence the mechanisms underlying cognitive impairment and the effectiveness of pharmacological treatments [116–118]. People on PD also experience unique stressors such as increased self-management responsibilities and less frequent contact with healthcare teams [114,119] (Griva et al., 2016; Walker et al., 2015) that might require specialized approaches tailored to their needs, routines, and care settings. Future studies into cognitive management should thus explicitly include PD populations, ideally through stratified designs or PD-specific trials, to evaluate the feasibility, acceptability, and effectiveness of interventions for this group. Qualitative research exploring the lived experience of cognitive challenges in PD are also warranted to offer important insights that can inform intervention development.

### Predialysis

Compared to the dialysis population, we found that considerably fewer interventions have been studied to address cognitive impairment in individuals with predialysis CKD, reflecting a major gap in the literature despite growing recognition that cognitive decline often begins in the early stages of kidney disease. Among the eight pharmacological interventions identified, most mirrored those studied in dialysis patients, targeting anemia, vascular dysfunction, uremic toxin accumulation, acidosis, and neurotransmitter dysfunction. However, these interventions were largely evaluated in single studies with minimal replication. Only four nonpharmacological interventions were studied, consisting of light-intensity exercise [43–45] and nutritional counselling [46], with no studies exploring approaches such as cognitive rehabilitation, psychosocial interventions, or combined strategies. The scarcity of interventions in this group is notable, given the potential to prevent or delay cognitive deterioration before the onset of dialysis. Research in predialysis CKD should prioritize early, multimodal intervention trials targeting the known drivers of cognitive impairment—including vascular health, systemic inflammation, and lifestyle factors—and assess both cognitive and functional outcomes over time. Three large multidomain trials (FINGER, MAPT and PreDIVA) have been completed in the past several years to prevent cognitive impairment in non-CKD populations [120–122], with the FINGER trial showing that a multidomain lifestyle intervention can benefit cognition in elderly people with an elevated risk of dementia [120]. Such multimodal approaches should be studied in people with predialysis CKD to help mitigate early cognitive decline in this population.

### Outcome measurement

Our review highlights several considerations for cognitive outcome measurement across the various CKD subgroups. First, we found that brief cognitive screens like the MMSE and MOCA were the most commonly-used outcome measures in existing CKD research. While these tools offer feasibility due to their brief length, their further validation as outcome measures in CKD is needed, given minimal evidence of their responsiveness to change in other populations [123,124]. In addition, these brief tools do not provide a fulsome assessment of cognition, and do not always accurately reflect real-world functioning as they do not assess a person performing learned, habitual tasks in their usual environment. Although executive functioning is a major cognitive domain affected by CKD, we found it was only specifically measured in ten studies. Executive functions are integral to independent living and community functions, and in turn, related to improved life satisfaction, health, and wellbeing [125–127]. Including more outcome measures of executive functioning to replace or augment global cognitive assessments might help to improve detection of meaningful cognitive changes in the CKD population. We also found that most studies relied on objective cognitive outcome measures, with only a limited number of studies capturing subjective cognitive functioning as an outcome. Although subjective cognitive tools alone are vulnerable to reporting biases and errors, they can provide additional insight into the impacts of cognitive impairment on real-world functioning when paired with objective tests. Subjective measures can help contextualize cognitive test results, identify functionally meaningful impairments, and capture concerns that might otherwise go undetected—particularly in early or subtle stages of cognitive decline (Jessen et al., 2014; Rabin et al., 2017). Such discrepancies can inform hypotheses about compensatory strategies, fatigue, mood, or contextual barriers that influence cognitive function (Vaughan et al., 2020). Integrating both perspectives is therefore essential for developing interventions that are not only statistically effective, but clinically relevant and aligned with patient priorities. As such, subjective cognitive assessments should be viewed as a critical complement to objective testing, and future intervention studies in the CKD population should incorporate both objective and subjective outcome measures to gain a more holistic understanding of treatment impact and real-world cognitive change.

### Strengths and limitations

This review has several methodological strengths. We followed the gold-standard JBI guidance [22] on scoping review conduct to maximize its quality and thoroughness, and both the protocol and the final manuscript adhere to the PRISMA-SCr reporting guidelines [24]. Our review used a comprehensive and systematic literature search refined by an information specialist. We also conducted duplicate full-text screening and data extraction of eligible articles. The limitations of this review include those inherent to scoping review methodology, such as a lack of critical appraisal of included articles, which makes it impossible to draw conclusions about the effectiveness of the interventions discussed. However, scoping review methodology lays the necessary groundwork for future systematic reviews by identifying research gaps and charting a course for knowledge advancement. Further, due to the large number of initial search results, and resource limitations among our team, we were unable to perform full duplication of title and abstract screening as is recommended by JBI scoping review guidelines. Not using two independent reviewers for all screening and selection poses a limitation through possible screening errors of one person. However, we conducted inter-rater validation for a subset of articles to ensure consistency among our screeners before independent screening was undertaken. We also excluded non-English studies from the review, which may have omitted relevant articles that reported results in other languages and may limit the generalizability of our findings to non-English populations.

## Conclusion

Research into interventions to improve cognitive performance for people with chronic kidney disease has primarily focused on the hemodialysis population and on investigating ESAs, frequent or prolonged dialysis, and exercise, although there has been an increase in the amount and scope of research activity in this area in recent years. Notable research gaps include the lack of research into promising nonpharmacological approaches such as cognitive rehabilitation and multimodal approaches; a lack of research into interventions targeting the diverse purported mechanisms of cognitive impairment in CI, such as cardiovascular disease, polypharmacy and depression; and a lack of research into cognitive interventions for people on peritoneal dialysis. Future research should continue to target a broader range of purported pathophysiological mechanisms of cognitive impairment in CKD; use RCTs and other robust methodologies to further establish efficacy; investigate cognitive rehabilitation approaches; include patient perspectives to improve the uptake and implementation of exercise training in cognitively impaired populations; and investigate interventions to promote cognitive functioning in PD.

## Supporting information

S1 Table PRISMA-ScR checklist. (PDF)

S1 File Data extraction sheet. (XLSX)

## Abbreviations

CKD: chronic kidney disease
CI: cognitive impairment
JBI: Joanna briggs institute
PRISMA-ScR: preferred reporting items for systematic reviews and meta-analyses – scoping reviews
